# Novel effects of acute COVID‐19 on cardiac mechanical function: Two case studies

**DOI:** 10.14814/phy2.14998

**Published:** 2021-08-27

**Authors:** Jyotpal Singh, Lanishen Bhagaloo, Eric Sy, Andrea J. Lavoie, Payam Dehghani, Patrick Neary

**Affiliations:** ^1^ Faculty of Kinesiology and Health Studies University of Regina Regina Saskatchewan Canada; ^2^ Gateway Alliance Medical Clinic Regina Saskatchewan Canada; ^3^ Faculty of Medicine University of Saskatchewan Saskatoon Saskatchewan Canada; ^4^ Department of Critical Care Saskatchewan Health Authority Regina Saskatchewan Canada; ^5^ College of Medicine University of Saskatchewan Regina Saskatchewan Canada; ^6^ Department of Cardiology Prairie Vascular Research Inc Saskatchewan Health Authority Regina Saskatchewan Canada

**Keywords:** cardiac cycle timing, cardiac dysfunction, case report, COVID‐19, heart performance index

## Abstract

The spread of the novel coronavirus 2019 (COVID‐19) has caused a global pandemic. The disease has spread rapidly, and research shows that COVID‐19 can induce long‐lasting cardiac damage. COVID‐19 can result in elevated cardiac biomarkers indicative of acute cardiac injury, and research utilizing echocardiography has shown that there is mechanical dysfunction in these patients as well, especially when observing the isovolumic, systolic, and diastolic portions of the cardiac cycle. The purpose of this study was to present two case studies on COVID‐19 positive patients who had their cardiac mechanical function assessed every day during the acute period to show that cardiac function in these patients was altered, and the damage occurring can change from day‐to‐day. Participant 1 showed compromised cardiac function in the systolic time, diastolic time, isovolumic time, and the calculated heart performance index (HPI), and these impairments were sustained even 23 days post‐symptom onset. Furthermore, Participant 1 showed prolonged systolic periods that lasted longer than the diastolic periods, indicative of elevated pulmonary artery pressure. Participant 2 showed decreases in systole and consequently, increases in HPI during the 3 days post‐symptom onset, and these changes returned to normal after day 4. These results showed that daily observation of cardiac function can provide detailed information about the overall mechanism by which cardiac dysfunction is occurring and that COVID‐19 can induce cardiac damage in unique patterns and thus can be studied on a case‐by‐case basis, day‐to‐day during infection. This could allow us to move toward more personalized cardiovascular medical treatment.

## Graphical abstract

Daily assessments of mechanical cardiac function provides insights to impairments and potential mechanisms in cardiac function following COVID‐19. A young participant with no previous medical history showed altered cardiac function which varied from day to day, and this damage was sustained for up to 3 weeks, whereas an older participant showed acute cardiac damage which was sustained for 3 days before returning to normal.

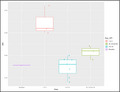

## 1 INTRODUCTION

In December 2019, the city of Wuhan, Hubei, China, reported a number of viral pneumonia cases of unknown origin (Huang, Wang, et al., 2020). The cause was later found to be severe acute respiratory syndrome coronavirus 2 (SARS‐CoV‐2), the virus that causes the novel coronavirus 2019 (COVID‐19) and can cause severe cardiac damage. As of April 23, 2021 there were more than 145 million cases globally with more than 3 million deaths (Dong et al., May 2020). This has presented unique challenges to the healthcare system and requires the embracement of new technologies to determine physiological mechanisms of injury with data acquired simply and easily.

There is sufficient evidence to suggest the association of cardiac injury with SARS‐CoV‐2 infection. However, underlying medical conditions can often exacerbate the damage and as such, thorough evaluation of medical history can provide greater insight into the disease progression. A meta‐analysis found that hypertension, cardiac disease, cerebrovascular disease, and diabetes were the most prevalent co‐morbidities, with hypertension and cardiac cerebrovascular disease being higher in severe patients (Li et al., 2020), and research is available to show the elevation of cardiac biomarkers, including troponins, C‐reactive protein, and N‐terminal pro‐brain natriuretic peptide (Guo et al., 2020; Lippi et al., 2020; Tian et al., 2020). Mechanical cardiac damage is now being studied more prominently in those COVID‐19 patients. Recent research shows that SARS‐CoV‐2 can infect cardiomyocytes through angiotensin‐converting enzyme 2 (ACE2) receptors and can impair cardiomyocyte contractility by breaking down sarcomeres and inducing cardiomyocyte cell death (Bailey et al., 2020). From 74 patients with confirmed COVID‐19, it was found that 41% of these patients had dilated right ventricles and 27% had right ventricular (RV) impairment (Mahmoud‐Elsayed et al., 2020). Furthermore, RV dysfunction was found to be associated with increased levels of D‐dimer and C‐reactive protein (Mahmoud‐Elsayed et al., 2020). These studies, therefore, suggest that when assessing cardiac function in COVID‐19, the mechanical cardiac function must always be considered. Specifically, the cardiac cycle timing events such as systole, diastole, isovolumic timing, and the rapid ejection period (REP). The assessment of these events will help to better understand the physiological mechanisms implicated in cardiac dysfunction.

**FIGURE 1:**
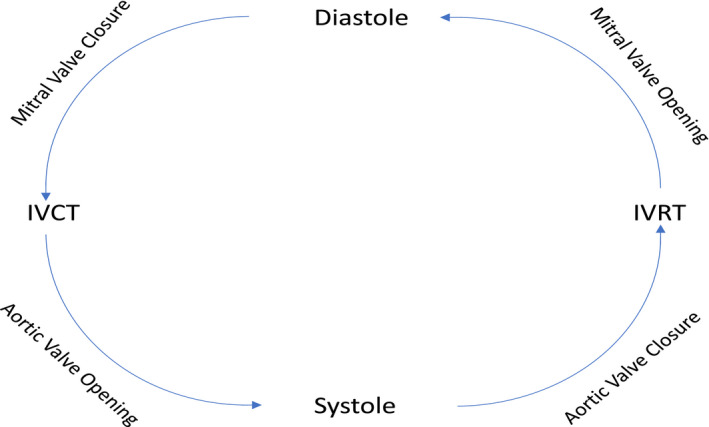
Aortic and mitral valve activity at cardiac cycle events. IVCT, isovolumic relaxation time; IVRT, isovolumic relaxation time

The mechanical aspects that control cardiac function can be represented by Wiggers Diagram (Mitchell & Wang, 2014). At the closure of the mitral valve, there is a period of increased ventricular pressure as the pressure (and volume) of the ventricle must be enough to cause the aortic valve to open. This period is known as the isovolumic contraction time (IVCT). Once the aortic valve opens, this leads to a phase known as systole, or the ejection period, at which blood rushes out of the aorta and induces systemic circulation. Once the aortic pressure overcomes the ventricular pressure, the aortic valve closes, and the left atrium fills with oxygenated blood from the lungs. This period is known as isovolumic relaxation time (IVRT), and once the atrial pressure overcomes the ventricular pressure, the mitral valve opens, leading to what is known as diastole. Diastole can be further subdivided into three more phases, known as rapid filling, slow filling, and atrial systole. It is important to note that IVCT and IVRT are sometimes considered part of systole and diastole, respectfully, but when assessing echocardiograms, these are generally considered as separate phases. For example, some studies calculate ejection time (systolic time) as the period following IVCT but before IVRT (Figure 1; Biering‐Sørensen et al., 2016). Previous research supports the reliability and validity of using transcutaneous force sensors based on a linear accelerometer, similar to the cardiac sensor used in the current study, to record precordial cutaneous vibrations taken from the sternum to assess cardiac mechanical function (Bombardini et al., Nov. 2011; Neary et al., 2011; Teckchandani et al., 2020).

The purpose of this study was twofold: (1) to present two case studies that showed that cardiac damage can manifest in different ways; and (2) to show the potential of assessing cardiac changes on a day‐to‐day basis using a cardiac sensor that can provide great insight into cardiac dysfunction and potentially help patient treatment utilizing personalized medical therapy.

## 2 MATERIALS AND METHODS

Prior to testing, this study was approved by the University of Regina Research Ethics Board (REB#2020‐073), and the participants signed an informed consent form. They were also asked general questions related to their physical activity level, medications, caffeine and alcohol consumption, sleep, and history of mild traumatic brain injury. A non‐invasive cardiac sensor (LLA Recordis^TM^; LLA Technologies) was used to record the mechanical function of the heart. Research has previously shown that this sensor can be used to observe changes in cardiac function in acute hypoxia (Teckchandani et al., 2020). This includes the assessment of cardiac timing (Bombardini et al., 2011) and contractility (Teckchandani et al., 2020), and this same analytical technique has been used to observe changes in the cardiac cycle timing parameters (Neary et al., 2011). The cardiac sensor was snapped into a single adhesive ECG gel‐electrode and placed 1 cm above the xiphoid process on the sternum of the chest over the skin. The participant was in a restful supine position, with a pillow under the head for comfort, for ~1‐min prior to turning the cardiac sensor on. The cardiac data were collected for 1 min. Symptoms were recorded each day prior to beginning data collection, with all data collected at approximately the same time of day. Data were recorded at symptom onset, with a COVID‐19 positive test result coming in the following 2 days.

The data were collected at 500 Hz and the raw signal was then analyzed on a beat‐to‐beat basis to process the cardiac cycle timing and amplitude signals. Specifically, a 1st order Butterworth bandpass filter with a low cut‐off frequency of 1 Hz and high cut‐off frequency of 30 Hz filter was applied to smooth the signal and adjust for the baseline wandering. Following this pre‐processing method, an in‐house, independent (proprietary) algorithm (LLA Technologies Inc) to assess the extracting features was applied to the waveform signal. The assessment was consistent with previous research (Crow et al., 1994; Neary et al., 2011). This resulted in the assessment of beats that have similar morphological features and thus ensured that all noise signals were removed from the analysis.

This analysis included the fiducial points of the cardiac cycle which included the mitral valve closure (MVC), aortic valve opening (AVO), aortic twist (ATT), aortic systole, REP, aortic valve closure (AVC), ventricular untwisting, mitral valve opening (MVO), and twist force (TWF). After the extraction of morphological features, temporal features were calculated (milliseconds, ms). This included diastole (MVC − MVO timing), systole (AVO − AVC timing), IVCT (MVC − AVO), IVRT (AVC − MVO), and end of rapid ejection (REP). Heart rate (AVO*_n_*_+1_ − AVO*_n_*) was calculated in beats per minute. Furthermore, the amplitude of the specific cardiac signals can be assessed as well by quantifying the magnitude of the acceleration (milligravity, mG) at the sternum (Neary et al., 2011). As the data collection period lasts for 1 min, the number of beats assessed is dependent on the heart rate.

Knowing the timing values for systole, IVCT and IVRT, heart (myocardial) performance indices can be calculated (Biering‐Sørensen et al., 2016). Specifically, the systolic performance index (SPI) was calculated as IVCT/systole, the diastolic performance index (DPI) was calculated by IVRT/systole, and the myocardial or heart performance index (HPI) was calculated as (IVCT+IVRT)/systole. These parameters can be beneficial to understand the fitness level of the heart (Biering‐Sørensen et al., 2016). Furthermore, these values can change depending on patient age and whether the patient exhibits a history of adverse cardiac events (Biering‐Sørensen et al., 2015).

All 1‐min data collected during a single testing period was presented as mean ± SD. To better understand the relative standard deviation during specific periods of elevated symptoms, the coefficient of variation (CV) was calculated for these times (CV = SD/mean).

## 3 RESULTS

Both participants are male (26 and 63 years old for participants 1 and 2, respectively). Both participants are regular caffeine consumers and intake limited alcohol. Furthermore, both participants exercise regularly as well. See Table 1 for further demographic information for both participants.

**TABLE 1:** Participant demographics

Parameter	Participant 1	Participant 2
Age (years)	26	63
Sex	Male	Male
Height (cm)	178	175
Mass (kg)	82	70
Daily caffeine intake (cups)	3	2
Weekly alcohol intake (#Drinks)	0	1–2
Hours of sleep per day	6	6
Hours of exercise per day	0.5	1–2
Estimated VO_2_max (ml kg^−1^ min^−1^)^a^	48	50
Medical conditions	NA	High cholesterol
History of mild traumatic brain injury	NA	5 total in lifetime, all before age 25 years

^a^ VO_2_max was based on the Heart Rate Ratio Method using the baseline data (Uth et al., 2004).

### 3.1 Symptoms for Participant 1

**FIGURE 2:**
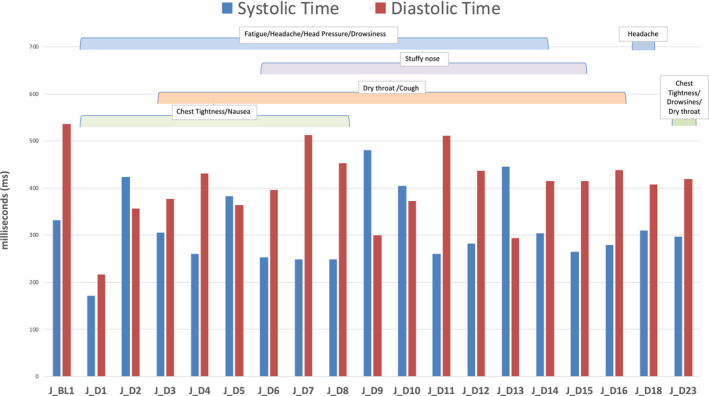
Systole and diastole timing intervals in Participant 1.

Participant 1 stated the presence of chest tightness, excessive fatigue, excessive drowsiness, heavy nausea, and excessive headache and pressure buildup in their head (Figure 2). The chest tightness developed into a “stabbing” feeling on day 2 post‐symptomatic onset, and the chest tightness remained elevated until day 8, while day 23 presented with elevated chest tightness again. Headache was present from day 1 to day 14 and again on day 18. Head pressure was present until day 14 and symptoms of nausea persisted until day 8. Fatigue was present until day 14, as was drowsiness, which was also elevated on day 23. With respect to respiratory symptoms, the participant exhibited dry throat from day 3 to day 14, and again on day 23, a stuffy nose from day 6 to day 13, and coughing persisted from day 3 to day 13.

### 3.2 Cardiac cycle timing parameters and TWF for Participant 1

The acute, symptomatic 23‐day period for Participant 1 showed varying results. Figure 2 and Table 2 document the cardiac events, and fortunately in our data collection, we had their baseline cardiac function assessed previously in the healthy state, and prior to their positive COVID‐19 test. This baseline data collection was completed using the same LLA Recordis™ sensor 6 months prior while the participant was healthy. Diagnosis was confirmed using the SARS‐CoV‐2 RNA polymerase chase reaction/nucleic acid amplification test (PCR/NAAT) in a certified provincial medical laboratory, and this participant had contracted the UK variant B.1.1.7 (20I/501Y.V1). When positive for COVID‐19 and symptomatic, Participant 1 showed prolonged impairments in all parameters of the cardiac cycle, including reductions in IVCT which did not return to the baseline level even on day 23. Interestingly, IVRT reductions were sustained as well, although on day 15, IVRT was back to baseline and this was also when IVCT was closest to baseline level. However, the IVCT and IVRT impairments returned the following day 15. Furthermore, the cardiac performance indices were also elevated on day 15, as systole appears shortened which resulted in an HPI of 0.52.

**TABLE 2:** Cardiac cycle timing parameters and twist force for Participant 1

Assessment	Heart rate (bpm)	Systolic time (ms)	Diastolic time (ms)	IVCT (ms)	IVRT (ms)	Rapid ejection time (ms)	Twist force (mG)	HPI	SPI	DPI
Baseline	61 ± 7	334 ± 66	472 ± 64	41 ± 16	96 ± 18	85 ± 21	10 ± 2	0.44 ± 0.12	0.13 ± 0.07	0.32 ± 0.13
Day 1	119 ± 7	168 ± 30	202 ± 39	25 ± 8	85 ± 18	29 ± 4	20 ± 2	0.61 ± 0.14	0.14 ± 0.06	0.52 ± 0.09
Day 2	70 ± 5	388 ± 59	355 ± 99	25 ± 4	66 ± 13	72 ± 13	14 ± 3	0.24 ± 0.04	0.06 ± 0.01	0.18 ± 0.04
Day 3	80 ± 5	309 ± 23	349 ± 38	24 ± 5	71 ± 8	60 ± 13	8 ± 2	0.30 ± 0.03	0.08 ± 0.02	0.23 ± 0.04
Day 4	82 ± 17	255 ± 41	423 ± 112	28 ± 7	67 ± 14	87 ± 11	12 ± 1	0.38 ± 0.08	0.12 ± 0.04	0.26 ± 0.06
Day 5	71 ± 4	390 ± 60	352 ± 61	22 ± 8	75 ± 15	87 ± 5	13 ± 2	0.26 ± 0.07	0.06 ± 0.02	0.20 ± 0.06
Day 6	82 ± 19	277 ± 57	364 ± 92	32 ± 11	79 ± 17	61 ± 15	12 ± 3	0.43 ± 0.13	0.13 ± 0.06	0.29 ± 0.09
Day 7	70 ± 10	238 ± 45	540 ± 85	40 ± 5	73 ± 9	71 ± 10	15 ± 2	0.47 ± 0.06	0.17 ± 0.04	0.32 ± 0.07
Day 8	76 ± 7	252 ± 51	424 ± 69	32 ± 6	79 ± 11	84 ± 10	15 ± 2	0.46 ± 0.10	0.13 ± 0.04	0.33 ± 0.08
Day 9	68 ± 2	478 ± 51	305 ± 53	29 ± 10	92 ± 14	105 ± 11	17 ± 2	0.26 ± 0.05	0.06 ± 0.02	0.19 ± 0.04
Day 10	68 ± 3	446 ± 71	345 ± 67	28 ± 5	79 ± 11	73 ± 15	16 ± 2	0.25 ± 0.07	0.07 ± 0.02	0.18 ± 0.06
Day 11	71 ± 15	278 ± 53	505 ± 148	37 ± 6	85 ± 17	77 ± 19	15 ± 3	0.45 ± 0.07	0.14 ± 0.03	0.31 ± 0.06
Day 12	73 ± 17	267 ± 87	473 ± 155	32 ± 10	81 ± 19	84 ± 13	12 ± 2	0.49 ± 0.30	0.14 ± 0.10	0.34 ± 0.20
Day 13	71 ± 3	436 ± 47	286 ± 69	31 ± 9	86 ± 22	66 ± 20	19 ± 2	0.31 ± 0.08	0.07 ± 0.03	0.21 ± 0.06
Day 14	77 ± 5	377 ± 69	292 ± 65	26 ± 8	83 ± 13	63 ± 5	17 ± 2	0.30 ± 0.09	0.07 ± 0.03	0.23 ± 0.07
Day 15	78 ± 20	265 ± 40	436 ± 135	43 ± 5	93 ± 14	81 ± 20	20 ± 2	0.52 ± 0.07	0.17 ± 0.03	0.35 ± 0.06
Day 16	72 ± 13	297 ± 60	429 ± 102	30 ± 9	80 ± 13	75 ± 17	13 ± 2	0.38 ± 0.08	0.11 ± 0.04	0.28 ± 0.07
Day 18	75 ± 6	322 ± 47	377 ± 59	23 ± 8	77 ± 8	83 ± 20	14 ± 2	0.32 ± 0.09	0.08 ± 0.04	0.24 ± 0.05
Day 23	73 ± 12	337 ± 69	429 ± 135	29 ± 9	79 ± 15	66 ± 14	15 ± 3	0.34 ± 0.11	0.09 ± 0.04	0.25 ± 0.08

Abbreviations: DPI, diastolic performance index; HPI, heart performance index; IVCT, isovolumic relaxation time; IVRT, isovolumic relaxation time; SPI, systolic performance index.

Observing the results on day 1 post‐symptom onset, there was a rapid increase in heart rate to 119 beats min^−1^, along with a reduction in systolic, diastolic, rapid ejection, IVCT, and IVRT timing (ms). However, because IVRT did not decrease to the same extent as IVCT, the decrease in systole resulted in a large increase in DPI (0.52). Furthermore, compared to the healthy baseline TWF (10 mG), day 1 post‐symptom onset resulted in a TWF of 20 mG (100% increase) suggesting an increase in ventricular stress. The changes on day 2 are further suggestive of cardiac impairments. While the heart rate was more similar to baseline during the infectious period, there was a large increase in systole (388 ms) from baseline which was greater than diastole (355 ms). Considering that IVCT (25 ms) remained lowered and IVRT (66 ms) decreased further, the HPI was drastically decreased on day 2 (0.24). Baseline (pre‐COVID) HPI was 0.44, implying a 45% reduction in HPI. Collectively, these results imply that this individual had both systolic and diastolic dysfunction. Systole was longer than diastole on days 5, 9, 10, 13, and 14, implying that pulmonary artery pressure may be elevated at these periods (Bombardini et al., 2011). Furthermore, the standard deviation of the heart rate was always very minimal at these periods between days 5 and 14. Thus, there may be further autonomic dysfunction at these periods, and assessment of heart rate variability may have provided further insight. Diastole and IVCT timings were also impaired in comparison to baseline on day 23, and while the performance indices are generally a stable value with minimal deviations, the participant showed a SD of 0.10 for HPI and a range of 0.24–0.61 from the day‐to‐day assessments. Although speculative, this implies that there may be potential cardiac adjustments and remodeling occurring to adapt to the ventricular stress. CV for SPI (healthy baseline = 0.52) and DPI (healthy baseline =0.42) was reduced until day 12, while HPI displayed varying CV responses. Therefore, this participant showed prolonged systolic and diastolic changes, likely the result of elevated pulmonary pressure and excessive ventricular stress which can lead to heart failure, cardiac remodeling, and fitness impairments.

### 3.3 Symptoms for Participant 2

Participant 2 also had their baseline data assessed previously in the healthy state, and prior to the confirmed positive COVID‐19 test. Diagnosis was confirmed using the SARS‐CoV‐2 RNA PCR/NAAT in a certified provincial medical laboratory, and this participant also had the UK variant B.1.1.7 (20I/501Y.V1). Participant 2 presented with a cough that persisted until day 4 and began to progressively improve thereafter. Muscle and joint aches lasted until day 5, while fatigue/tiredness, and general malaise lasted for 10 days. Chills improved on days 3–4, and sleep improved on day 8. Headaches during the initial 4 days were “moderate” intensity, lasted all day and this contributed to the disrupted sleep pattern and reduced sleep quality. Headaches improved on day 10 and the participant regularly took Advil (400 mg x 3 per day) and cannabidiol (~100 mg daily^−1^) in an oil tincture sublingually under the tongue for 1–2 min before swallowing. Finally, smell and taste were not impaired initially during the first 4–5 days, with only a slight reduction from day 5 to 10, but returning back to normal by day 20. The participant returned to mild aerobic stationary cycling exercise (100 beats min^−1^) slowly, and progressively (days 12–19), and increased exercise intensity and duration back to normal by day 20 (145–160 beats min^−1^; ~80%–90% predicted HRmax). Participant 2 experienced no long‐lasting symptoms, with the most excessive symptoms in the first 5 days.

### 3.4 Cardiac cycle timing parameters and TWF for Participant 2

**FIGURE 3:**
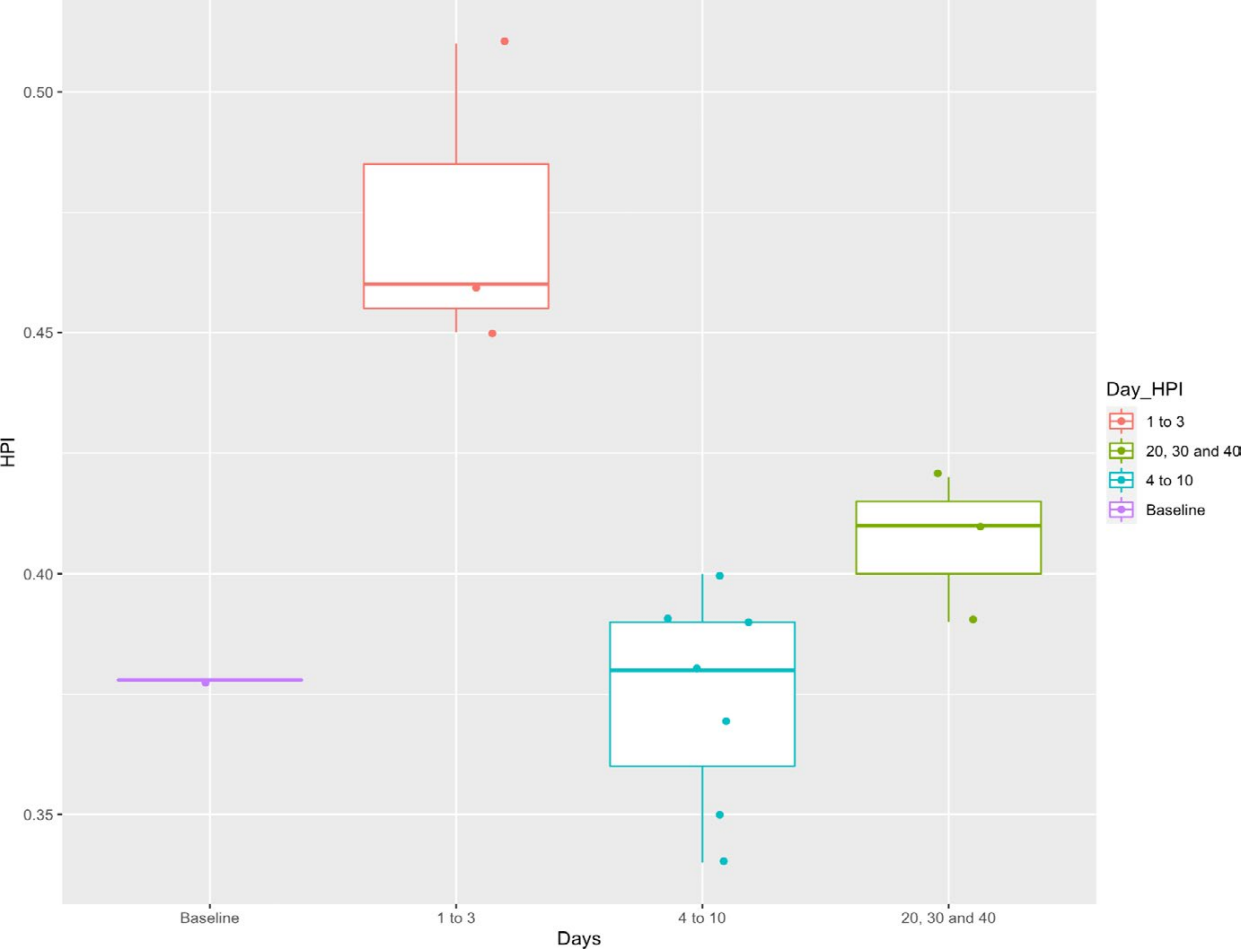
Heart performance index for Participant 2

This baseline data collection was completed using the same LLA Recordis™ sensor 2 months prior to COVID‐19 for Participant 2. Participant 2 showed acute impairments in some parameters of the cardiac cycle (Table 3; Figure 3). Specifically, as compared to their healthy baseline data, Participant 2 showed increases in cardiac performance indices which returned to normal on day 4 post‐symptom onset. During the first 3 days post‐symptom onset, systole was decreased (20%–23%). Because IVRT and IVCT did not decrease to the same extent, there was an elevated HPI (0.51, 0.46, and 0.45 on days 1, 2, and 3, respectively). This implies elevated cardiac dysfunction and stress. At baseline (pre‐COVID), HPI was at 0.38 ± 0.26 (CV = 0.26), and when symptoms were at their worst (day 2), this value increased to 0.51 ± 0.10 (CV = 0.15), suggesting increased stress on the heart, and similar to values reported on patients with congestive heart failure (Biering‐Sørensen et al., 2015). From day 4 onwards, the participant exhibited cardiac function similar to their healthy state, including a normal HPI. Figure 3 shows the box plots for the greatest change that occurred between days 1 and 3 post‐symptom onset and the jittered points reflect that the HPI values within those periods do not deviate excessively from the mean. Participant 2 had increased symptoms for the first 5 days, with the worst symptoms on day 2. Considering the systolic time, the baseline value was measured at 373 ± 72 ms (CV = 0.19 ms), and on day 2 this dropped to 291 ± 16 ms (CV = 0.05 ms) with the average of the first 5 days following symptom onset being 314 ms (average CV = 0.13 ms; range of 0.05–0.18 ms). There was a decrease in the relative standard deviation when symptoms are the highest in Participant 2 as shown by the lowered CV values. Again, this demonstrates that heart contraction variability was reduced in the acute COVID‐19 stages.

**TABLE 3:** Cardiac cycle timing parameters and twist force for Participant 2

Assessment	Heart rate (bpm)	Systolic time (ms)	Diastolic time (ms)	IVCT (ms)	IVRT (ms)	Rapid ejection time (ms)	Twist force (mG)	HPI	SPI	DPI
Baseline	48 ± 1	373 ± 72	740 ± 74	37 ± 3	104 ± 18	109 ± 31	27 ± 3	0.38 ± 0.10	0.10 ± 0.02	0.28 ± 0.08
Day 1	49 ± 3	291 ± 16	791 ± 67	36 ± 5	112 ± 17	117 ± 31	29 ± 4	0.51 ± 0.08	0.12 ± 0.02	0.38 ± 0.07
Day 2	61 ± 3	286 ± 53	554 ± 89	34 ± 3	99 ± 26	76 ± 6	32 ± 5	0.46 ± 0.14	0.12 ± 0.02	0.35 ± 0.12
Day 3	55 ± 2	299 ± 22	662 ± 45	35 ± 2	99 ± 19	77 ± 4	30 ± 4	0.45 ± 0.09	0.12 ± 0.01	0.33 ± 0.08
Day 4	48 ± 3	353 ± 62	768 ± 101	37 ± 4	96 ± 17	90 ± 19	30 ± 4	0.38 ± 0.09	0.10 ± 0.02	0.27 ± 0.07
Day 5	47 ± 3	347 ± 61	786 ± 121	33 ± 3	107 ± 22	103 ± 30	29 ± 4	0.40 ± 0.11	0.10 ± 0.01	0.31 ± 0.10
Day 6	49 ± 3	339 ± 53	765 ± 75	36 ± 4	90 ± 19	82 ± 18	30 ± 4	0.37 ± 0.09	0.11 ± 0.01	0.27 ± 0.08
Day 7	49 ± 4	331 ± 61	767 ± 112	33 ± 4	97 ± 18	97 ± 28	31 ± 4	0.39 ± 0.09	0.10 ± 0.02	0.29 ± 0.07
Day 8	47 ± 2	376 ± 76	750 ± 78	35 ± 3	95 ± 17	118 ± 32	30 ± 4	0.34 ± 0.08	0.09 ± 0.02	0.25 ± 0.07
Day 9	49 ± 2	339 ± 62	732 ± 73	37 ± 7	97 ± 18	82 ± 13	28 ± 3	0.39 ± 0.10	0.11 ± 0.03	0.29 ± 0.07
Day 10	49 ± 3	348 ± 53	757 ± 70	31 ± 4	91 ± 14	97 ± 26	33 ± 3	0.35 ± 0.07	0.09 ± 0.02	0.26 ± 0.06
Day 20	52 ± 2	329 ± 41	699 ± 58	35 ± 3	93 ± 19	96 ± 18	27 ± 3	0.39 ± 0.07	0.11 ± 0.01	0.28 ± 0.07
Day 30	49 ± 1	317 ± 17	771 ± 38	33 ± 3	102 ± 13	85 ± 6	24 ± 3	0.42 ± 0.06	0.10 ± 0.01	0.32 ± 0.05
Day 40	47 ± 2	340 ± 64	806 ± 76	35 ± 3	105 ± 15	114 ± 34	30 ± 4	0.41 ± 0.09	0.10 ± 0.02	0.31 ± 0.07

Abbreviations: DPI, diastolic performance index; HPI, heart performance index; IVCT, isovolumic relaxation time; IVRT, isovolumic relaxation time; SPI, systolic performance index.

## 4 DISCUSSION

This study documents novel findings, and to our knowledge we present for the first time the effects of diagnosed COVID‐19 on the cardiac mechanical function when assessed daily during both the symptomatic and asymptomatic continuous monitoring periods. Furthermore, we present data that include baseline cardiac cycle function in the normal healthy (pre‐COVID) state prior to a positive diagnosis of COVID‐19. The above case studies suggest that the systolic and diastolic phases of the cardiac cycle can be affected differently in different individuals infected by COVID‐19, verifying that COVID‐19 infection can be unique and individualized. Participant 1 showed large increases and decreases in the cardiac performance indices (HPI, SPI, and DPI), suggesting that there was sustained cardiac dysfunction when observing the isovolumic times, that is, IVCT and IVRT. Furthermore, the increase in diastolic time compared to systolic time implies elevated pulmonary artery pressure (Bombardini et al., 2011). Based on the observed changes in these specific cardiac timing events, myocardial stress appeared to be remarkably high in Participant 1, and this is supported by the symptoms the participant experienced which included chest tightness and pain with the highest symptoms on days 1–8. What was very unique to this study is the extension of diastole over systole. Indeed, this can imply potential valvular dysfunction or the inability of the cardiac myocytes to maintain adequate ventricular pressure. This may be a unique finding in this study as the impaired cardiac cycle timing intervals can be reflective of the excessive stress on the pulmonary artery. Increases in the heart rate due to exercise can result in shortened diastole; however, in a diseased state, this phenomenon requires more research.

Participant 2 did not show sustained prolonged cardiac dysfunction; however, the changes were evident during the acute period (days 2–5). This included elevated cardiac performance indices (HPI) during the first 3 days from symptom onset and greatest reductions in systolic time during this acute stage as well.

Collectively, these results showed three important findings. First, a younger individual (26 years old) showed unique cardiac dysfunction due to COVID‐19 even with no previous medical history or pathology. Second, a younger individual (26 years old) did not have their cardiac function return to their normal healthy (pre‐COVID) state even post 3 weeks. Third, acute cardiac dysfunction related to the virus does not necessarily indicate that chronic long‐term effects will be maintained, even in older individuals (60 years+), and they too can recover quickly (as demonstrated in Participant 2). Our ability to monitor cardiac function on a day‐to‐day basis has provided new insight into how the virus affected the heart during acute COVID, and how heart mechanics recovers over the 2–4 weeks post‐symptoms. This provides a unique opportunity to assess those that present with long‐lasting symptoms post COVID‐19, such as those with long COVID, to determine whether cardiac effects are present in long COVID, and to examine the physiological mechanism(s) implicated in the long‐term effects of the virus.

Changes in cardiac performance indices have been shown to be associated with cardiac damage. In a study that examined the original severe acute respiratory syndrome virus (SARS) on cardiac function during the acute phase and then after 30 days in 46 patients, it was found that left ventricle myocardial performance and IVRT were increased while cardiac output was decreased acutely (Li, Cheng, et al., 2003), implying that acute SARS pneumonia does result in greater ventricular stress. Specifically, an acute HPI of 0.42 was found during the acute phase compared to 0.33 when assessed 30 days later. Furthermore, when stratifying for those who required mechanical ventilation, an HPI of 0.51 was found at the acute assessment period compared to 0.32 for these same individuals 30 days later (Li, Cheng, et al., 2003). Elevated levels of HPI and IVRT and decreased systolic time have been shown in COVID‐19 patients (Kaya et al., 2021). However, the data presented for Participant 1 suggest there can be drastic changes in the performance indices and cardiac cycle timing events, and thus, these variations suggest that daily changes can provide more information as to the mechanistic changes occurring during the symptomatic period and during recovery. Specifically, many of the changes seen in the cardiac parameters can be due to the dynamic daily changes in cardiac function, thus implying that these non‐invasive, day‐to‐day measurements can provide fresh insights into the mechanisms of the cardiac dysfunction.

While many different methods of assessing mechanical cardiac function exist, the implementation of the heart performance indices can be utilized in a manner to observe the systolic activity as a function of the isovolumic periods. Reductions in LV systolic function result in increased time required for the myocardial myocytes to allow for ventricular pressure to match aortic pressure, thereby increasing IVCT and reducing the systolic time as ventricular pressure cannot be maintained (Biering‐Sørensen et al., 2015; Carluccio et al., 2012). This reduction in LV function also results in a decrease in diastolic function, thus impairing IVRT. However, if IVCT and IVRT lengthened and systolic time is reduced, then HPI increases (Biering‐Sørensen et al., 2015). Increases in HPI can therefore detect cardiac dysfunction, independent of associating systolic or diastolic dysfunction, thus, SPI and DPI are calculated to provide the finer and more subtle details (Biering‐Sørensen et al., 2015) for the cardiac dysregulation. The potential impairments of ACE2 receptors following SARS‐CoV‐2 infection may help explain some of these cardiac impairments.

### 4.1 Potential for ACE2 involvement

Severe acute respiratory syndrome coronavirus 2 does bind to the ACE2 receptor to gain entry into cells. The spike protein of the SARS and SARS‐CoV‐2 virus is used for attachment to target cells at the S1 unit and the S2 unit facilitates viral entry (Hoffmann et al., 2020; Huang et al., 2020), followed by priming which is completed by the type II transmembrane protease TMPRSS2 in SARS (Glowacka et al., 2011), and similarly, SARS‐CoV‐2 (Hoffmann et al., 2020). SARS‐CoV can enter and damage cells by attaching to and interacting with the ACE2 receptor (Li, Moore, et al., 2003), and it was found that SARS‐CoV‐2 also uses the ACE2 receptor for entry (Hoffmann et al., 2020) as the receptor‐binding domain strongly interacts with the ACE2 receptor (Chen et al., 2020). This is followed by the reduction of ACE2 mRNA and increases in ADAM metallopeptidase domain 17 gene expression (Gross et al., 2020). This is important to note as ACE2 can also act to degrade angiotensin (Ang) II to Ang (1–7). Ang II induces a wider array of cardiac damage as it can stimulate the Ang II type 1 receptor (AT1) to induce vasoconstriction and increase blood pressure, potentially lead to fibrosis, and induce inflammation and excessive oxidative stress (South et al., 2020). Moreover, Ang (1–7) can stimulate the Mas receptor, resulting in vasodilatory and anti‐inflammatory responses (South et al., 2020).

Thus, while elevated ACE2 levels can help to better maintain vasomotor control and a lower sympathetic tone, elevated levels of ACE2 are generally seen in individuals with cardiac complications. The upregulation of ACE2 is important as it does allow for cardioprotective effects which otherwise would not be possible. However, given that SARS‐CoV‐2 uses ACE2 receptors as its entry point, it does create a paradoxical situation in which those with heightened levels of ACE2 due to any potential cardiovascular impairments are more susceptible to cardiac injury because of their greater ACE2 expression. Considering that older individuals would also have elevated levels of Ang II which can become hyperactive following COVID‐19 infection, the over‐exaggerated inflammatory response (“cytokine storm”) can be a direct indicator of the high severity risk in older individuals (AlGhatrif et al., 2019). Indeed, a case‐cohort study that analyzed plasma concentrations of ACE2 and potential determinants of plasma ACE2 levels, it was found that ACE2 was associated with increased risk of total deaths, and increased risk of cardiac complications, including the incident of heart failure, myocardial infarction, stroke, and diabetes in non‐COVID patients (Narula et al., 2020). Interestingly, Ang II does weaken the baroreceptor sensitivity which will induce vasoconstriction, inflammation, and other physiological responses that promote hypertension, and the formation of ACE2 will counteract these effects, including the enhancement of baroreceptor sensitivity (South et al., May 2020).

### 4.2 Previous mechanical cardiac research

Community‐acquired pneumonia has also been shown to induce changes in cardiac function. A cohort of 545 patients with community‐acquired pneumonia underwent cardiovascular assessment with results showing the presence of atrial fibrillation in 9.5% of the population, and those with atrial fibrillation showed higher indexed left atrial area and concentric LV hypertrophy (Cangemi et al., 2019). A transthoracic echocardiography of 46 patients with the original SARS virus was assessed acutely and after 30 days (Li, Cheng, et al., 2003). Their results showed that ejection time, IVRT, and the LV index of myocardial performance were significantly greater at baseline compared to day 30 follow‐up, while LV ejection fraction was acutely lower in patients that required mechanical ventilation (Li, Cheng, et al., 2003). Tricuspid annular plane systolic excursion (TAPSE) has been shown to be reduced in patients with community‐acquired pneumonia (Yıldırım et al., 2015). In COVID‐19 patients, it was found that TAPSE was one of the variables negatively associated with troponin levels (Szekely et al., 2020). Pneumonia appears to have an impairing effect on mechanical heart function, and both SARS‐CoV and SARS‐CoV‐2 also seem to cause ventricular damage. Therefore, our study adds further evidence that mechanical cardiac damage can occur due to COVID‐19, and this can present unique dysfunctions from one individual to the next, suggesting the importance of daily monitoring using a cardiac sensor such as that used in this study.

## 5 CONCLUSION AND LIMITATIONS

While COVID‐19 is known to induce cardiac damage, there is still more research required to understand the progression of the cardiac damage induced by the virus. Here, a novel method to assess daily cardiac function was presented with two case studies, each showing uniquely different cardiac dysfunctions. Participant 1 (26 years of age) showed changes related to impairments in the cardiac cycle potentially due to elevated pulmonary artery pressure and did not return to the healthy baseline level assessed prior to SARS‐CoV‐2 infection. Participant 2 (63 years of age) showed acute cardiac dysfunction which returned to baseline on day 4 and thereafter fluctuated around at healthy baseline levels. This data, therefore, showed that even younger individuals such as Participant 1 can have sustained cardiac damage due to COVID‐19. Daily monitoring of cardiac activity can provide insight into the overall physiological impairments which may otherwise go unnoticed, and the knowledge of having healthy baseline data (pre‐COVID) showed the direct changes induced by the virus. Although data are presented on only two participants and the results can only be generalized to these individuals, this study provides valuable information about the physiological mechanism(s) and the importance of assessing cardiac function on a day‐to‐day basis. The LLA Recordis™ is similar to a heart rate monitor and can be used daily for recording but provides information about cardiac mechanics, that is, cardiac cycle timing events. Lack of blood sample analysis and echocardiography comparisons limits the study and would have helped to confirm the extent of cardiac damage. Statistics were limited to descriptive data.

## DISCLOSURE

The authors have no conflict of interest in this project.

## AUTHOR CONTRIBUTION

Jyotpal Singh, Lanishen Bhagaloo, and J. Patrick Neary conceived and designed the experiments and contributed to the writing and revising of the manuscript. Eric Sy, Andrea J. Lavoie, and Payam Dehghani contributed to the understanding and analyses of the data, and the revision of the manuscript. All authors have seen and approved the final manuscript.

## Data Availability

The authors confirm that the data supporting the findings of this study are available within the article.
